# Fully Biodegradable Poly(hexamethylene succinate)/Cellulose Nanocrystals Composites with Enhanced Crystallization Rate and Mechanical Property

**DOI:** 10.3390/polym13213667

**Published:** 2021-10-25

**Authors:** Siyu Pan, Zhaobin Qiu

**Affiliations:** State Key Laboratory of Chemical Resource Engineering, Beijing University of Chemical Technology, Beijing 100029, China; 2020200319@grad.buct.edu.cn

**Keywords:** cellulose nanocrystals, poly(hexamethylene succinate), crystallization

## Abstract

Through a common solution and casting method, low contents of cellulose nanocrystals (CNC) reinforced biodegradable poly(hexamethylene succinate) based composites were successfully prepared for the first time. CNC homogeneously dispersed in PHS matrix at low loadings, showing no obvious aggregation. PHS/CNC composites showed high thermal stability as PHS. As a heterogeneous nucleating agent, CNC promoted the crystallization of PHS under both nonisothermal and isothermal crystallization conditions. In addition, the higher the CNC content, the faster the crystallization of PHS/CNC composites. The heterogeneous nucleating agent role of CNC was directly confirmed by the crystalline morphology study; moreover, the crystal structure of PHS remained unmodified despite the presence of CNC. As a reinforcing nanofiller, CNC also improved the mechanical property of PHS, especially the Young’s modulus and yield strength. In brief, low contents of CNC may improve both the crystallization and mechanical property of PHS, providing an easy method to tune the physical property and promote the wider application of biodegradable polymers.

## 1. Introduction

Biodegradable polymers are of great importance from a sustainability viewpoint [1,2]. Some aliphatic polyesters, such as poly(butylene succinate) (PBS) and poly(ethylene succinate) (PES) are typical biodegradable polymers and have recently become an important focus in both academic and industrial fields [3,4,5,6,7,8,9,10]. Similar to other biodegradable polymers, such as poly(butylene adipate-*co*-terephthalate) (PBAT) and poly(L-lactide) (PLLA), the two most-known, commercially available ones, PBS has also been industrialized in Japan, China, and Thailand since in the last three decades [11]. Similar to PBS and PES, poly(hexamethylene succinate) (PHS) is also a biodegradable polyester with a chemical structure of (-OCH_2_CH_2_CH_2_CH_2_CH_2_CH_2_O_2_CCH_2_CH_2_CO-)_n_.

PHS is a semicrystalline polyester with a low glass transition temperature of about −40 °C and an equilibrium melting point (*T*_m_°) of 77 °C [12]. Franco and Puiggail found the fastest crystallization rate of PHS occurred at −3 °C; moreover, a crystallization transition from regime II to III took place at 27 °C. PHS showed negative spherulites when it crystallized from the melt [12]. Puiggail et al. also determined the crystal structure of PHS as a monoclinic unit cell with *a* = 1.612 nm, *b* = 1.464 nm, *c* = 1.440nm, and *β* = 38.6° [13]. To adjust the physical property and extend the practical application, some PHS based copolymers and nanocomposites have recently been studied [14,15,16,17,18]. For instance, Wei et al. synthesized several phosphorus-containing PHS based copolyesters with bulky pendent group. The noncrystallizable bulky comonomer units, which mainly resided outside the crystal lattice of PHS, seriously retarded the crystallization of PHS [14]. In previous research, we studied the effect of molecular weight on the crystallization behavior of poly(hexamethylene succinate-*co*-3 mol% ethylene succinate) (PHES). PHES with high molecular weight crystallized more slowly than that with low molecular weight at the same crystallization temperature [16]. In addition, we further synthesized poly(hexamethylene succinate-*co*-6 mol% butylene succinate) (PHBS), a novel PHS based copolyester. The *T*_m_° of PHBS was slightly lower than that of PHS; furthermore, the copolymer crystallized more slowly than the homopolymer although they crystallized through the same crystallization mechanism [17]. In the literature, Puiggail et al. described how they prepared two types of PHS/clay nanocomposites with intercalated structure through a solution and casting method. Both types of clay (C30B and C20A) enhanced the thermal stability and promoted the crystallization of PHS; moreover, the increase in the crystallization rate was greater for the PHS/C20A nanocomposite [18].

Biodegradable, biobased, highly crystalline, and rod-like cellulose nanocrystals (CNC), derived from a renewable resource, may reinforce and improve the mechanical property of polymer matrix; moreover, CNC can also promote the crystallization of semicrystalline polymers, especially biodegradable polymers, as an efficient nucleating agent [19,20,21,22,23]. In the literature, some fully biodegradable polymer/CNC composites have been extensively studied, including PLLA, PBAT, PBS, PES, poly(β-hydroxybutyrate) (PHB), poly(ε-caprolactone) (PCL), poly(ethylene adipate) (PEA), and poly(butylene succinate-*co*-butylene adipate) (PBSA) [24,25,26,27,28,29,30,31,32,33,34,35].

To the best of our knowledge, PHS/CNC composites have not been reported so far. The reasons to perform this research are as follows. On the one hand, unlike other biodegradable polymers, such as PLLA, PBAT, PBS, and PES, PHS has received less research attention in both academic and industrial fields. As there are so many methylene groups in the chemical repeating unit, PHS may show good flexibility and toughness, similar to those of PCL. However, its other mechanical property, such as Young’s modulus and tensile strength need to be improved from a viewpoint of practical application. On the other hand, the slow crystallization rate of PHS also affects the physical property and polymer processing efficiency. Therefore, in order to improve the mechanical property and accelerate the crystallization of PHS, PHS/CNC composites with low contents of CNC were prepared in this work through a solution and casting method. The dispersion of CNC in PHS matrix was investigated first; furthermore, the influence of CNC on the thermal stability, crystallization behavior, and mechanical property of PHS/CNC composites was studied in detail. The results revealed that CNC promoted the crystallization and improved the mechanical property of PHS, providing a convenient method to improve the physical property and promote the practical application of fully biodegradable polymer/CNC composites. The novelty of this research is outlined as follows. First, fully biodegradable PHS/CNC composites were prepared and reported for the first time. Second, even low contents of CNC (not higher than 1 wt%) could remarkably accelerate the crystallization of PHS as an efficient nucleating agent. Third, a balanced mechanical property could be achieved for the PHS/CNC composite when CNC content was 0.5 wt%, which may find many potential applications as a packaging material.

## 2. Experimental Section

### 2.1. Materials

PHS (*M*_n_ = 3.42 × 10^4^ g/mol, *M*_w_ = 5.83 × 10^4^ g/mol) was synthesized by our laboratory. CNC (5–20 nm in diameter and 50–200 nm in length) was bought from Shanghai ScienceK Nanotechnology Co., Ltd.

PHS/CNC composites were prepared through a solution and casting process. N, N-dimethylformamide was chosen as a cosolvent. The detailed preparation process was similar to that described in previous work [34]. The composites were abbreviated as PHS/CNC0.25, PHS/CNC0.5, and PHS/CNC1, respectively, with the number indicating CNC content in wt%.

**Scheme 1:**
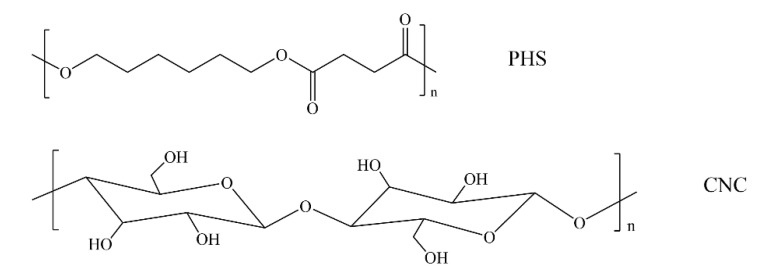
The chemical structures of PHS and CNC.

Scheme 1 shows the chemical structures of PHS and CNC.

### 2.2. Characterization

The dispersion of CNC in the composites were observed with a scanning electron microscope (SEM) (JEOL, JSM-7800) (Tokyo, Japan). After the hot-pressed films were fractured in liquid nitrogen with a tweezer, the newly acquired fractured surfaces of PHS and PHS/CNC composites were coated with gold for observation.

The thermal stability study of PHS and PHS/CNC composites was monitored by thermogravimetric analysis (TGA) (TA Instruments Q50) (New Castle, DE, USA).

The crystallization behavior of PHS and PHS/CNC composites was investigated under nitrogen atmosphere with differential scanning calorimetry (DSC) (TA Instruments Q100) (New Castle, DE, USA). The mass was about 4 to 5 mg.

The crystalline morphology of PHS and PHS/CNC composites was investigated with polarized optical microscopy (POM) (Olympus BX51) (Tokyo, Japan) equipped with a hot stage (Linkam THMS 600) (Surrey, UK).

The wide angle X-ray diffraction (WAXD) profiles of PHS and PHS/CNC composites were acquired on an X-ray diffractometer (Rigaku Ultima IV) (Tokyo, Japan) from 5° to 45° at a rate of 5°/min.

The tensile mechanical property of PHS and PHS/CNC composites was measured with a universal tensile testing machine (UTM5205XHD) at 20 mm/min at room temperature. At least, three specimens were used to obtain the average data.

## 3. Results and Discussion

### 3.1. Dispersion of CNC in PHS Matrix and Thermal Stability Study

**Figure 1:**
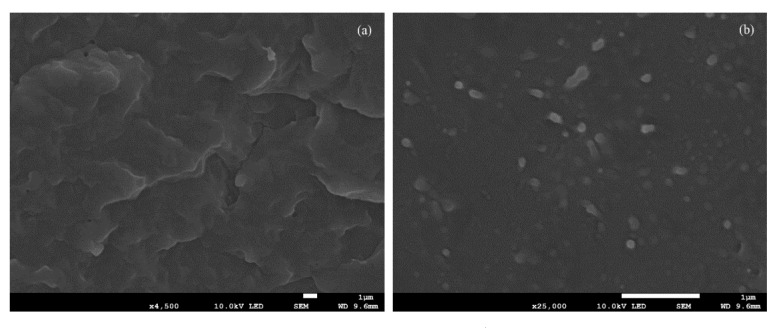
SEM images of (**a**) PHS and (**b**) PHS/CNC1.

It is well-known that the homogeneous dispersion of CNC in polymer matrix is essential to enhance the physical properties of polymer/CNC composites [19,20,21,22,23]. In this research, the dispersion of CNC was first observed with SEM in PHS matrix. Parts a and b of Figure 1 show the SEM images of the fractured surfaces of PHS and PHS/CNC1, respectively. The unmodified PHS demonstrated a rough fractured surface, indicative of its good toughness. As clearly shown in Figure 1b, CNC rods randomly dispersed in PHS matrix, showing no obvious aggregation, even CNC content was 1 wt%. As a result, a homogeneous dispersion of CNC in PHS matrix was verified for PHS/CNC1. In brief, when CNC content was 1 wt% and below, CNC may homogeneously disperse in PHS matrix.

**Figure 2:**
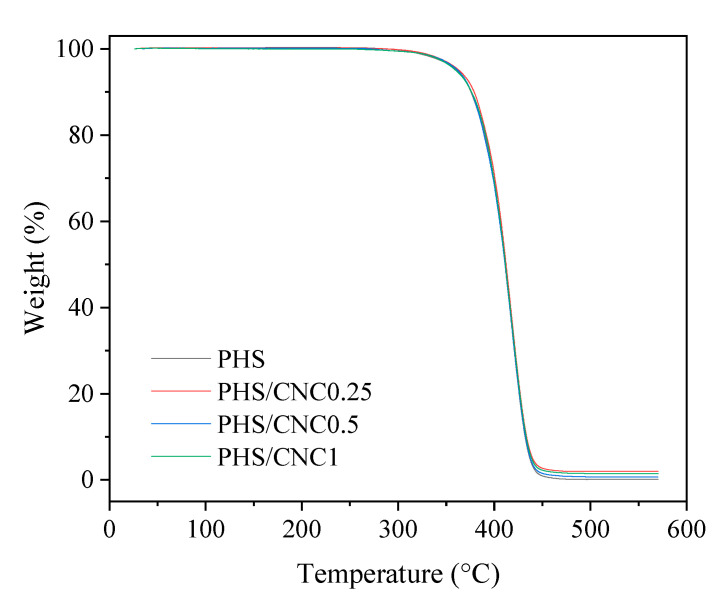
TGA traces of PHS and PHS/CNC composites.

The thermal stability study is important from both polymer processing and practical application viewpoints. TGA was used in this research to measure the thermal stability of PHS and PHS/CNC composites, which were heated at 20 °C/min under nitrogen atmosphere. Figure 2 displays the TGA results. All samples showed the similar thermal degradation process, i.e., one-step decomposition process, suggesting that CNC did not affect the thermal degradation mechanism. From Figure 2, the degradation temperature at 5 wt% weight loss (*T*_d_) was directly measured to be 360.8 °C for PHS. In the composites, the *T*_d_ values varied slightly between 359.7 and 363.1 °C, demonstrating that PHS/CNC composites had the similar high thermal stability as PHS.

### 3.2. Crystallization Behavior Study

**Figure 3:**
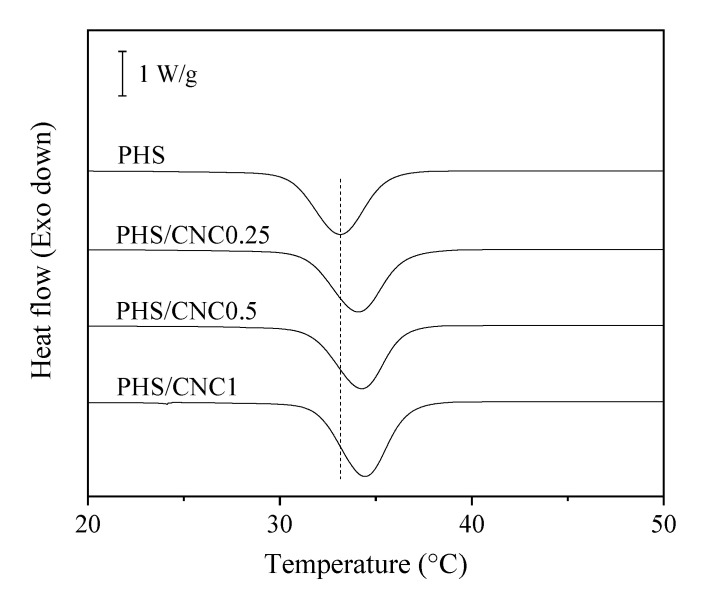
DSC cooling traces of PHS and PHS/CNC composites at 5 °C/min.

The crystallization behavior of PHS/CNC composites was further studied and compared with that of PHS. Figure 3 displays the DSC cooling traces of PHS and PHS/CNC composites at 5 °C/min after the previous thermal history of each sample was first erased. Under such a nonisothermal crystallization condition, both PHS and the composites showed the well-defined crystallization exothermic peaks. The melt crystallization temperature (*T*_cc_) appeared at 33.2 °C for PHS and slightly increased to 34.1, 34.3, and 34.5 °C for the composites, with the increase in CNC content. Such a result indicated that the melt crystallization of PHS was promoted to some extent by the nucleation effect of CNC. In addition, the melt crystallization enthalpy varied slightly between 60.0 and 64.2 J/g for PHS and its composites; consequently, CNC only slightly affected the crystallinity of PHS.

**Figure 4:**
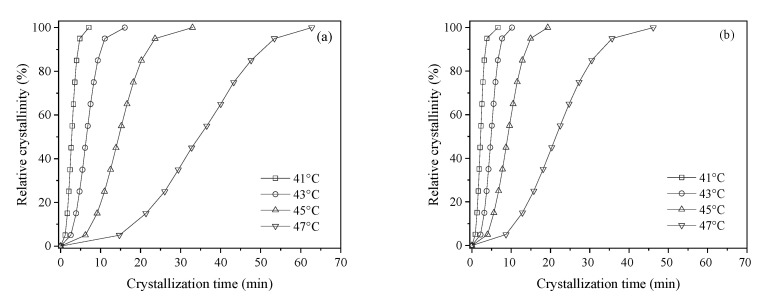

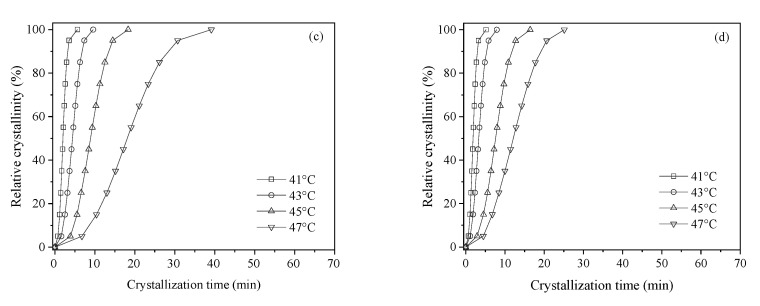
Plots of relative crystallinity versus crystallization time of (**a**) PHS, (**b**) PHS/CNC0.25, (**c**) PHS/CNC0.5, and (**d**) PHS/CNC1.

The isothermal crystallization kinetics of PHS and PHS/CNC composites was also investigated in a crystallization temperature (*T*_c_) range of 41 to 47 °C after eliminating previous thermal history. Under such an isothermal crystallization condition, the evolution of heat flow with time was recorded and analyzed for both PHS and its composites. Figure 4 illustrates the development of relative crystallinity, calculated from the ratio of the integrated area from onset crystallization time to crystallization time *t* to that of the whole crystallization exotherm from onset crystallization time to end crystallization time with the DSC software, versus crystallization time for all samples at indicated *T*_c_s. Conversely, with the increase in *T*_c_, crystallization time gradually prolonged for each sample, regardless of CNC content, indicative of a slow crystallization rate. From a polymer crystallization viewpoint, such a result is reasonable, as the driving force gradually decreases due to the increased *T*_c_, thereby making the nucleation and crystal growth more difficult at a small degree of supercooling. On the other hand, at the same *T*_c_, crystallization time remarkably was shorter in the composites than in PHS, indicating again that CNC accelerated the crystallization of PHS as a heterogeneous nucleating agent.

**Figure 5:**
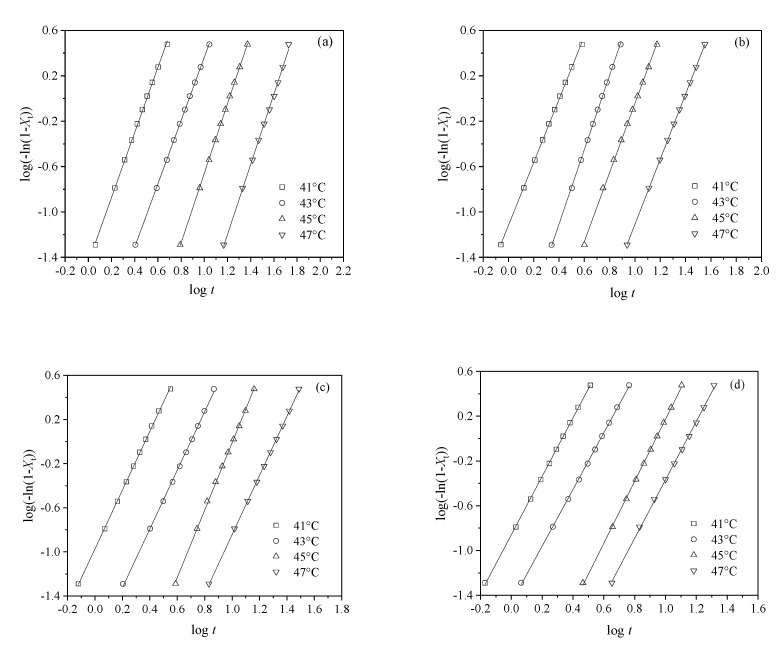
Avrami plots of (**a**) PHS, (**b**) PHS/CNC0.25, (**c**) PHS/CNC0.5, and (**d**) PHS/CNC1.

The isothermal crystallization kinetics of PHS and PHS/CNC composites was further analyzed by the classical Avrami method as follows:

1 − *X*_t_ = exp(−*kt^n^*) (1)

where *X*_t_ is relative crystallinity formed at crystallization time (*t*), *n* is the Avrami exponent, and *k* is the crystallization rate constant [36,37,38,39]. Figure 5 depicts the Avrami plots for all samples, from which the experimental *X*_t_ data were well fitted by the Avrami equation with different Avrami parameters.

Table 1 lists the summary of Avrami parameters for PHS and PHS/CNC composites. Regardless of *T*_c_ and CNC content, *n* slightly varied between 2.5 and 3.3. The average *n* (2.85) was close to 3, indicating that PHS/CNC composites and PHS crystallized through the same crystallization mechanism within the investigated *T*_c_ range, i.e., an athermal nucleation and three-dimensional truncated sphere growth mechanism [40].

For each sample, the *k* values gradually increased with the decrease in *T*_c_, indicating a shorter period of time required to complete crystallization. At the same *T*_c_, the *k* values increased with CNC content, suggesting that CNC promoted the crystallization of PHS.

To accurately explore the influence of *T*_c_ and CNC content, crystallization half-time (*t*_0.5_), the time to reach 50% of the final crystallinity, was used in this research. On the basis of the following equation, which is derived from the above Avrami equation, *t*_0.5_ was calculated.

*t*_0.5_ = (ln 2/*k*)^1/*n*^ (2)

**Figure 6:**
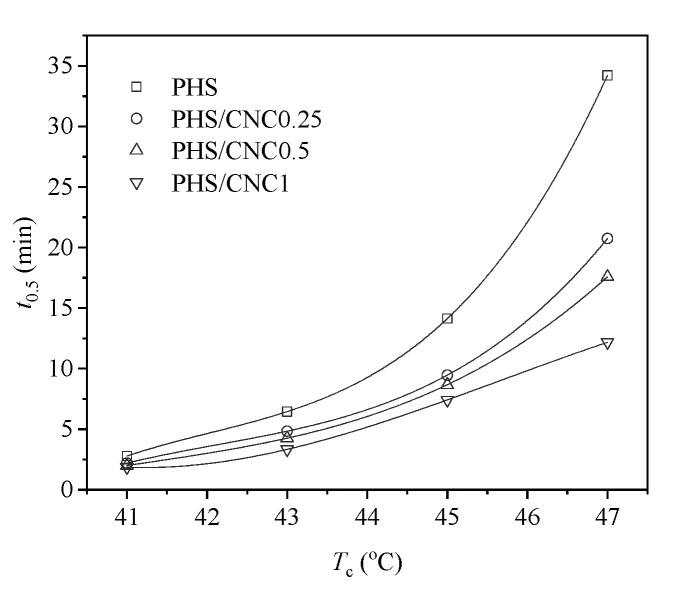
Plots of *t*_0.5_ versus *T*_c_ for PHS and PHS/CNC composites.

Figure 6 illustrates the plots of *t*_0.5_ versus *T*_c_ for PHS and PHS/CNC composites. On the one hand, *t*_0.5_ gradually decreased with the decrease in *T*_c_ for each sample, indicating a faster crystallization rate. For instance, *t*_0.5_ of PHS/CNC0.25 remarkably decreased from 20.7 to 2.2 min with decreasing *T*_c_ only from 47 to 41 °C. On the other hand, *t*_0.5_ was smaller in the composites than in PHS; moreover, at the same *T*_c_, *t*_0.5_ gradually decreased with increasing CNC content. For example, at the same *T*_c_ of 47 °C, *t*_0.5_ remarkably decreased from 34.2 min for PHS to 12.2 min for PHS/CNC1. Such a result clearly indicated that CNC promoted the crystallization of PHS as a nucleating agent.

**Figure 7:**
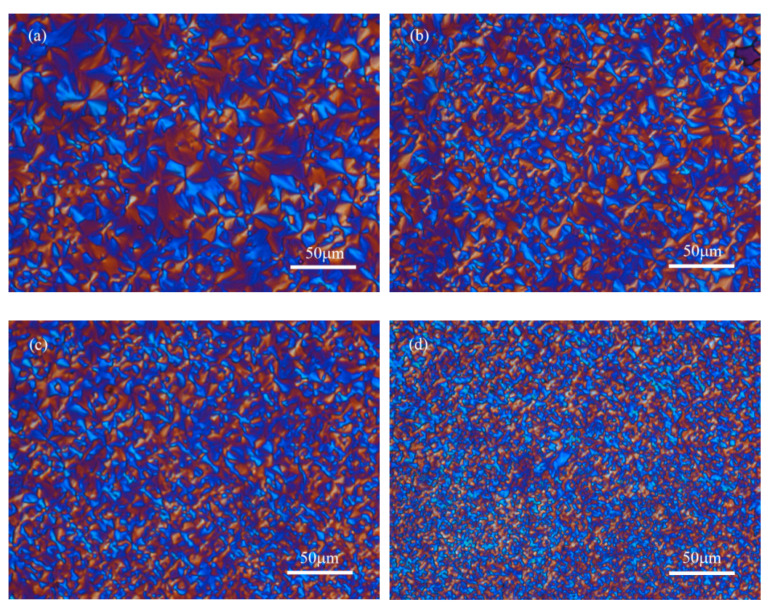
POM images of (**a**) PHS, (**b**) PHS/CNC0.25, (**c**) PHS/CNC0.5, and (**d**) PHS/CNC1 at 45 °C.

The above studies indicated that CNC promoted the crystallization of PHS in the composites as a nucleating agent. In this section, the crystalline morphology study was performed with POM. Figure 7 shows the POM images of PHS and PHS/CNC composites after crystallizing at 45 °C for sufficient time. Regardless of CNC content, spherulitic morphology was observed for each sample. From Figure 7, the number of PHS spherulites significantly increased with increasing CNC content; consequently, the size of PHS spherulites gradually decreased. The crystalline morphology variation in PHS spherulites confirms the nucleating agent effect of CNC, as the nucleation density of PHS spherulites remarkably increased due to the presence of CNC. In brief, the crystalline morphology directly provided the evidence of the nucleating agent effect of CNC.

**Figure 8:**
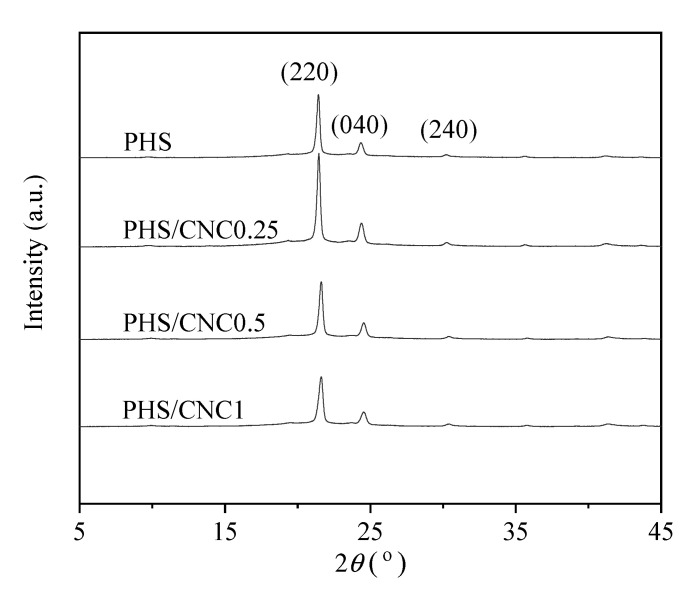
WAXD patterns of PHS and PHS/CNC composites.

Figure 8 depicts the WAXD profiles of PHS and PHS/CNC composites after they finished crystallization at 45 °C for 5 h. Three relatively strong diffraction peaks at 2*θ* of 21.5°, 24.4°, and 30.3°, corresponding to the (220), (040), and (240) planes of PHS, respectively, were observed for PHS and PHS/CNC composites [13]. For both PHS and its composites, they displayed the similar WAXD profiles, suggesting that CNC did not modify the crystal structure of PHS. In addition, from Figure 8, the crystallinity values were estimated to be about 55% for PHS, PHS/CNC0.5, and PHS/CNC1, respectively. In the case of PHS/CNC0.25, the crystallinity was slightly increased to about 60%. In sum, CNC did not obviously affect the crystallinity of PHS, especially at CNC content of 0.5 wt% and above.

### 3.3. Tensile Mechanical Property Study

**Figure 9:**
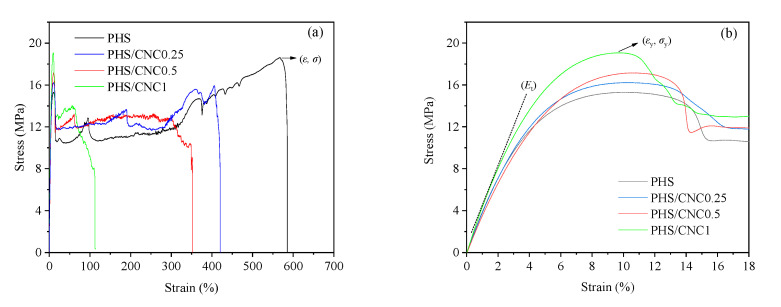
(**a**) whole and (**b**) initial stress-strain curves of PHS and PHS/CNC composites.

The effect of CNC on the tensile mechanical property of PHS was further studied in this section. Parts a and b of Figure 9 illustrates the whole and initial stress-strain curves of PHS and PHS/CNC composites, respectively. As illustrated in Figure 9, CNC obviously influenced the tensile mechanical property of PHS. The detailed mechanical property data are summarized in Table 2. With the increase in CNC content, the Young’s modulus (*E*_t_) gradually increased from 360.2 ± 10.6 MPa for PHS to 460.8 ± 14.5 MPa for PHS/CNC1, indicative of the reinforcing role of CNC. As also shown in Figure 9, the yielding behavior was found for both PHS and PHS/CNC composites at low strains, i.e., yield strain (*ε*_y_) of below 10%; moreover, the yield strength (σ_y_) obviously increased from 16.39 ± 0.01 MPa for PHS to 18.35 ± 0.51 MPa for PHS/CNC1, indicating again the the reinforcing role of CNC. Accordingly, the elongation at break (*ε*) gradually decreased. CNC did not apparently affect the tensile strength at break (*σ*) of PHS. From Table 2, when CNC content was 0.5 wt%, the composite showed a *E*_t_ of 430.9 ± 11.8 MPa, a *σ* of 16.87 ± 0.43 MPa, and a *ε* of 398.2 ± 32.4%. In the literature, poly(ε-caprolactone) (PCL) showed a *E*_t_ of 340 MPa, a *σ* of 20 MPa, and a *ε* of 300–1000%, while poly(butylene adipate) (PBA) showed a *E*_t_ of 170 MPa, a *σ* of 11–16 MPa, and a *ε* of 380–560% [41]. In addition, PBS showed a *E*_t_ of 300–500 MPa, a *σ* of 34 MPa, and a *ε* of 560% [41]. From the above data, the mechanical properties of PHS/CNC0.5 composite were comparable to those of PCL. The mechanical properties of PHS/CNC0.5 composite were superior to those of PBA, because the *E*_t_ of the former (430.9 ± 11.8 MPa) was significantly greater than that of the latter (170 MPa), while they had the comparable *σ* and *ε* values. In addition, the mechanical properties of PHS/CNC0.5 composite were inferior to those of PBS, because the *σ* of the former (16.87 ± 0.43 MPa) was obviously smaller than that of the latter (34 MPa) while they had the comparable *E*_t_ and *ε* values. Similar to PCL, PBA, and PBS, PHS/CNC composite may find potential applications in many fields, such as shopping bags, waste bags, mulch film, express packaging, take-out tableware, and some disposable items, since they have comparable mechanical properties.

In addition, it is interesting to compare the PHS/CNC composites with other biodegradable polymer/CNC composites. Han et al. recently studied PCL/CNC composites with CNC content from 1 to 10 wt% and also found increasing CNC content could increase the crystallization rate and improve the strength [32]. But in the present work, we found that even a low content of CNC could remarkably increase the crystallization and obtain a better balanced mechanical property between strength and toughness.

## 4. Conclusions

Biodegradable PHS/CNC composites containing low contents of CNC from 0.25 to 1 wt% were prepared through a solution and casting method. The dispersion of CNC in PHS matrix and the effect of CNC on the thermal stability, crystallization behavior, and mechanical property of PHS were extensively studied with SEM, TGA, DSC, POM, WAXD, and tensile tests. Through the SEM observation, CNC was found to homogeneously disperse in PHS matrix, showing no obvious aggregation. Both PHS/CNC composites and PHS showed a similar good thermal stability, with the decomposition temperature of about 360 °C. Under different crystallization conditions, CNC enhanced the crystallization of PHS. On the one hand, CNC slightly increased the melt crystallization temperature during a cooling process at 5 °C/min. On the other hand, CNC increased the isothermal crystallization rate of PHS in a temperature range of 41 to 47 °C. Increasing crystallization temperature gradually decreased the crystallization rates of both PHS/CNC composites and PHS; moreover, the higher the CNC content, the faster the crystallization rate of PHS/CNC composites. For instance, 1 wt% of CNC remarkably reduced the crystallization half-time of PHS from 34.2 to 12.2 min. The Avrami method well described the isothermal crystallization kinetics of PHS/CNC composites and PHS. Regardless of CNC content and crystallization temperature, the crystallization mechanism did not change. The spherulitic morphology revealed an obvious decrease in the size of PHS spherulites, providing direct evidence of the nucleating agent effect of CNC. In addition, CNC did not modify the crystal structure of PHS. As a reinforcing nanofiller, CNC improved the mechanical property of PHS, especially the Young’s modulus and yield strength. PHS/CNC composite with 0.5 wt% of CNC exhibited the comparable mechanical properties to those of other biodegradable polymers such as PCL, PBA, and PBS, showing a *E*_t_ of 430.9 ± 11.8 MPa, a *σ* of 16.87 ± 0.43 MPa, and a *ε* of 398.2 ± 32.4%. In sum, the crystallization and mechanical property of PHS may be improved by low contents of CNC, providing a suitable method to adjust the physical property and extend the wider practical application of biodegradable polymers.

## Figures and Tables

**Table 1:** Summary of Avrami parameters of PHS and PHS/CNC composites.

Samples	*T*_c_ (°C)	*n*	*k* (min^−*n*^)
PHS	41	2.9	3.58 × 10^−2^
	43	2.8	3.75 × 10^−3^
	45	3.1	1.88 × 10^−4^
	47	3.1	1.22 × 10^−5^
PHS/CNC0.25	41	2.8	7.61 × 10^−2^
	43	3.3	3.82 × 10^−3^
	45	3.0	8.19 × 10^−4^
	47	2.9	1.05 × 10^−4^
PHS/CNC0.5	41	2.7	1.07 × 10^−1^
	43	2.7	1.39 × 10^−2^
	45	3.1	8.58 × 10^−4^
	47	2.7	3.01 × 10^−4^
PHS/CNC1	41	2.6	1.38 × 10^−1^
	43	2.5	3.41 × 10^−2^
	45	2.8	2.53 × 10^−3^
	47	2.6	1.04 × 10^−3^

**Table 2:** Tensile mechanical property data of PHS and PHS/CNC composites.

Samples	Young’s Modulus (MPa)	Yield Strength (MPa)	Yield Strain (%)	Tensile Strength (MPa)	Elongation at Break (%)
PHS	360.2 ± 10.6	16.39 ± 0.01	9.16 ± 0.06	18.84 ± 0.46	560.0 ± 35.5
PHS/CNC0.25	386.8 ± 10.6	17.18 ± 0.36	9.18 ± 0.34	16.48 ± 0.28	406.1 ± 15.2
PHS/CNC0.5	430.9 ± 11.8	17.30 ± 0.15	9.67 ± 0.56	16.87 ± 0.43	398.2 ± 32.4
PHS/CNC1	460.8 ± 14.5	18.35 ± 0.51	9.48 ± 0.46	18.53 ± 0.31	88.6 ± 6.5

## Data Availability

The data presented in this study are available on request from the corresponding author.
